# Performance Analysis of Typical Data Fusion Algorithms for Inertial Measurement Arrays

**DOI:** 10.3390/mi17080969

**Published:** 2026-08-17

**Authors:** Ting Zhu, Zhenzhen Guan, Qiwen Wang, Wei Wu, Jingbei Tian

**Affiliations:** 1 School of Automation, Guangxi University of Science and Technology, Liuzhou 545006, China; t@imu.wiki (T.Z.); 20240202027@stdmail.gxust.edu.cn (Z.G.); 20240201024@stdmail.gxust.edu.cn (Q.W.); 20240201011@stdmail.gxust.edu.cn (W.W.); 2 Guangxi Key Laboratory of Logistics Unmanned Aircraft Technology for Transportation Industry, Liuzhou 545616, China; 3 Guangxi Low-Altitude Unmanned Aircraft Key Technologies Engineering Research Center, Liuzhou 545616, China

**Keywords:** MEMS gyroscope array, data fusion, Kalman filtering, weighted least squares, bias stability, power-on repeatability, root mean square error, IMU configuration

## Abstract

This paper investigates data fusion for multi-MEMS gyroscope arrays by comparing four methods: numerical averaging, weighted least squares, direct estimation Kalman filtering, and indirect estimation Kalman filtering. The state-estimation characteristics and observability of the two Kalman-filter models are also analyzed. The performance of the four methods is evaluated through controlled simulations, static experiments, dynamic turntable experiments, and array-size analysis. The simulation and static experimental results show that when the IMUs exhibit similar Allan bias-instability characteristics, the four methods yield relatively similar results in terms of bias instability. When the Allan bias-instability characteristics of the IMUs differ, numerical averaging provides poorer performance, whereas the other three methods yield comparable results in terms of bias instability. When different error components are present and exhibit conflicting trends, fixed weighting based on a single statistical indicator may lead to weight mismatch. In the dynamic experiment, the three-axis gyroscope and three-axis accelerometer measurements are separately processed using the same fusion method, and the resulting six-axis fused data are used as inputs to the PSINS inertial navigation system, with the final horizontal position drift adopted as a system-level performance metric. Under the tested dynamic conditions, the direct estimation Kalman filter achieves the smallest final horizontal position drift, followed by the indirect estimation Kalman filter, while both outperform numerical averaging and fixed-weight fusion. The array-size analysis shows that the performance gain gradually diminishes as the number of IMUs increases. The geometric knee point is located at approximately 21 IMUs, and the main performance-transition range is approximately 21–30 IMUs. Under the simulation and experimental conditions considered in this study, this range provides a favorable trade-off among fusion performance, computational burden, and array complexity.

## 1. Introduction

In 2003, Bayard et al. proposed the concept of a virtual gyroscope, in which four low-cost gyroscopes were arranged to form a gyroscope array, and Kalman filtering was employed to recursively obtain an optimal estimate from multiple gyroscope outputs [1]. In this way, a virtual gyroscope output with higher accuracy than that of a single gyroscope could be achieved. Their study verified the feasibility of inertial sensor array fusion through simulation and demonstrated significant potential for drift suppression under ideal correlation conditions. Building upon this array configuration, Xue et al. further explored optimal redundant structures for non-orthogonal MEMS inertial sensor arrays, demonstrating that careful geometric layout combined with a customized Kalman filter can effectively minimize non-orthogonal coupling errors [2]. Meanwhile, Zhuang et al. combined virtual gyro technology with an improved Sage–Husa adaptive Kalman filter to address the challenge of time-varying noise statistics in dynamic environments, achieving a reduction in angular random walk of approximately 88% [3]. In 2008, Chang et al. designed an integrated MEMS gyroscope array and adopted a two-stage optimal filtering structure to fuse the outputs of multiple gyroscopes [4]. In 2012, Jiang et al. introduced a first-order Markov process into the modeling of angular velocity signals from a six-gyroscope array and combined it with Kalman filtering to optimize the array output [5]. In 2014, Xue et al. further analyzed the dynamic performance of Kalman filter-based fusion for multiple MEMS gyroscopes. Experimental results showed that, under constant-rate conditions, the angular velocity error of the fused output from a six-gyroscope array was reduced by more than eight times compared with that of a single gyroscope [6]. In 2015, Yuan et al. compared the performance of a direct estimation Kalman model and a differential-estimation Kalman model in the fusion of a six-gyroscope array [7]. The results indicated that the direct estimation model achieved better error suppression under low-dynamic or constant-input conditions, whereas the differential-estimation model could weaken the influence of some individual sensor errors by constructing relative observations among sensors.

In addition to Kalman filtering, weight-assignment-based fusion methods constitute another important branch of IMU array data fusion. In 2012, Zhang et al. proposed a support-degree-based information fusion method for MEMS gyroscopes, in which a support-degree matrix was constructed and combined with weighted least squares to obtain an angular velocity estimate [8]. In 2016, Sun and Liu introduced an adaptive weighting strategy on the basis of support-degree fusion. By adjusting the influence of each sensor’s observation variance on the corresponding weight, the total variance of the fused target parameter was minimized [9]. For non-stationary motion scenarios, Lan et al. proposed an adaptive filtering method based on MEMS-IMU array data fusion, which effectively suppressed dynamic noise through multi-sensor collaboration and adaptive covariance tuning [10]. In 2017, Vaccaro and Zaki established a random drift correlation model among multiple low-cost gyroscopes based on Allan covariance and constructed a low-drift virtual gyroscope using an optimal linear combination method [11]. Their results showed that considering error correlations among sensors could further improve the rationality of weight assignment. Meanwhile, an in-depth analysis of correlation coefficients in MEMS gyroscope arrays revealed that inter-sensor error correlation significantly affects the accuracy improvement factor of the fused output, and that negative correlation can further enhance the improvement ratio [12]. In recent years, with the development of intelligent optimization algorithms, weighted fusion methods have gradually shifted from weight assignment based on empirical formulas or statistical characteristics to global weight optimization aimed at minimizing fusion error. In 2025, Zhu, Gao Peng, and colleagues proposed a weighted average fusion method for MEMS IMU arrays based on the fruit fly optimization algorithm, in which the optimal weights of gyroscopes and accelerometers were searched using the fruit fly optimization algorithm [13]. In the same year, Li et al. introduced the crested porcupine optimizer into inertial measurement array fusion, realizing adaptive weight allocation through swarm intelligence optimization, thereby improving array output accuracy and suppressing random noise [14]. Additionally, homogeneous sensor fusion optimization studies have further validated the effectiveness of adaptively adjusting sensor weights based on root mean square error, providing a practical reference for weight determination in engineering implementations [15].

In recent years, deep learning has also been introduced into inertial array fusion and integrated navigation error compensation, forming hybrid fusion frameworks that combine Kalman filtering with neural networks. In 2023, Miao Lin et al. proposed a gyroscope array fusion algorithm based on neural networks and Kalman filtering [16]. In their method, an LSTM-RNN was used to calculate the confidence of each gyroscope, and the confidence values, measurement data, and angular velocity estimated by Kalman filtering were jointly input into a BP neural network for fusion, thereby improving the robustness of the fusion algorithm in the presence of faulty gyroscopes. Furthermore, to address the online compensation of random errors in MEMS arrays, a hybrid filtering compensation algorithm combining wavelet threshold denoising with a BP neural network was proposed, which effectively suppressed the random errors of array outputs under complex operating conditions [17]. Zhu et al. proposed a combined method based on a long short-term memory network and Kalman filtering for MEMS gyroscope error compensation in random vibration environments. Their results showed that the combined method could effectively reduce the standard deviation of gyroscope errors and improve the smoothness of the compensated output [18]. In addition, in 2024, Xu et al. proposed a GRU-assisted adaptive Kalman filtering method to address the divergence of inertial navigation errors during GNSS outages, reflecting the development trend of neural-network-assisted filtering in the field of inertial navigation [19]. Meanwhile, Mi et al. leveraged deep learning to improve the performance of MEMS gyroscope arrays under unknown disturbances, demonstrating that a well-designed LSTM network can effectively model and compensate for complex error characteristics that are difficult to capture by traditional filtering methods [20].

In summary, the recent review literature on MEMS IMU array technology has systematically traced the technical evolution from basic numerical averaging and Kalman filtering to intelligent optimization and neural-network-assisted fusion, providing a comprehensive overview of the development trajectory in this field [21]. Existing studies have evolved from basic fusion methods such as numerical averaging, weighted least squares, and Kalman filtering to more complex fusion frameworks, including adaptive weighting, intelligent optimization-based weight assignment, and neural-network-assisted filtering. However, in practical engineering applications, basic fusion methods remain important because of their simple structure, low computational cost, and strong interpretability. In particular, for low-cost IMU arrays, it is still necessary to further compare the performance differences among numerical averaging, weighted least squares, and conventional Kalman filtering under the same experimental conditions, as well as to analyze the influence of the number of IMUs on fusion accuracy and system cost. Therefore, this paper focuses on the theoretical derivation and experimental investigation of the above three basic fusion methods, and further analyzes the trade-off between cost and accuracy with respect to the scale of the IMU array, aiming to provide a reference for the selection of IMU array fusion schemes in practical engineering applications.

In practical IMU-array design, increasing the number of IMUs can improve measurement stability through redundant observations; however, it also increases hardware cost, power consumption, system volume, data-processing requirements, and computational burden. Therefore, the selection of the array size should be regarded as a trade-off between accuracy improvement and engineering cost. Previous studies have investigated the optimal configuration of redundant inertial sensors by jointly considering navigation accuracy, reliability, system size, and cost, demonstrating that simply increasing the number of sensors may not represent the most effective engineering solution [22].

The performance of an IMU array depends not only on the number of sensors but also on the array configuration and data-fusion algorithm. He et al. improved the positioning accuracy of a ZUPT-aided pedestrian inertial navigation system using a differential-layout MIMU array, demonstrating that an appropriate array configuration can improve measurement and navigation performance [23]. Wu et al. proposed an array-based consensus strategy for a four-gyroscope array under unknown-input fluctuations and showed that appropriate error modeling, local estimation, and adaptive fusion can substantially suppress bias fluctuations without requiring a large array [24].

Based on these observations, this study introduces an incremental accuracy-gain criterion to evaluate the relationship between array size and fusion performance. Each additional IMU is treated as an incremental hardware input, while the corresponding reduction in bias stability represents the incremental accuracy gain. As the array size increases, the improvement contributed by each additional IMU may gradually decrease. When the incremental gain becomes sufficiently small, further expansion of the array may no longer be justified by the associated increase in hardware and computational costs.

To identify this transition objectively, a maximum-distance-based geometric criterion is adopted. After normalizing the array-size and STD(n) coordinates, the perpendicular distance from each data point to the straight line connecting the first and last points of the normalized curve is calculated. The point with the maximum perpendicular distance is selected as the transition point. This criterion provides a reproducible means of identifying the array size at which the balance between accuracy improvement and engineering cost begins to deteriorate.

## 2. Typical Data Fusion Algorithms and Principles

Data fusion refers to the comprehensive processing of redundant or complementary observations from multiple low-cost and low-precision IMUs through a specific algorithmic framework, thereby producing an optimal state estimate at the system level that exceeds the accuracy limit of any single independent unit. Its fundamental purpose is to suppress random noise using statistical methods, compensate for systematic errors, and improve system reliability and fault tolerance through hardware redundancy. In an inertial measurement array system, multiple IMUs measure the same external state variable at the same time. However, because the measurement accuracy of each IMU is different, their measured values also differ. Therefore, how to reasonably and effectively fuse the data from multiple IMUs to obtain more accurate and stable measurement results has become a key issue in data fusion technology.

### 2.1. Numerical Averaging Method

The averaging method is a basic and straightforward fusion approach. Its core idea is to simply sum the observations from multiple sensors and calculate their average value. It features simplicity, intuitiveness and high computational efficiency. For sensors with similar precision and minor errors, the averaging method can smooth out partial errors and improve system stability. Nevertheless, this method is only applicable when the measurement accuracy of each device is close.

In cases where the measurement accuracy varies across sensors, the precision of data sources differs greatly, or the interference noise of each sensor is inconsistent, the simple averaging method cannot distinguish such discrepancies. Consequently, individual data with large errors will degrade the fusion performance and reduce overall measurement accuracy.

Its formula is expressed as:

(1) $$\hat{x}=\frac{1}{n}\sum_{i=1}^{n}x_{i}$$

(2) $$\sigma_{\mathrm{avg}}^{2}=\frac{1}{n^{2}}\sum_{i=1}^{n}\sigma_{i}^{2}$$

### 2.2. Weighted Least Squares

The weighted least squares method is an improved fusion approach based on numerical averaging. Unlike simple averaging, it assigns different weights to sensors according to their measurement accuracy and noise level. Sensors with higher accuracy and lower noise variance receive larger weights, while less reliable sensors receive smaller weights. This weighting strategy reduces the influence of low-accuracy or high-noise measurements on the final estimate.

From an estimation perspective, when sensor measurements are mutually independent and their noise variances are known, weighted least squares provides an unbiased linear estimate with minimum variance. Its basic principle is to assign greater influence to more reliable measurements, thereby improving the accuracy and stability of the fused result.

However, the method requires prior knowledge of sensor noise statistics and is more suitable for static or quasi-static measurements. It has limited ability to describe temporal correlations in dynamic systems or suppress systematic errors such as bias, installation errors, and temperature drift. Therefore, it is mainly applicable to multi-sensor fusion problems in which the measurements are independent and their noise variances can be obtained through calibration or statistical analysis.

In the weighted least squares fusion model, the fused estimate can be expressed as the weighted sum of the measurements from all sensors:

(3) $$\hat{x}=\sum_{i=1}^{n}w_{i}x_{i}$$

where $w_{i}$ denotes the weight coefficient of the $\sum_{i=1}^{n}w_{i}=1$ sensor, satisfying the normalization condition, with $w_{i}\geq 0$ The weights are usually determined according to the variance or noise level of each sensor. Suppose that the measurement noise variance of each sensor is $\sigma_{i}^{2}$, and that the measurement noises are mutually independent.

To ensure the unbiasedness of the fused estimate, the weights should satisfy the normalization constraint. Since the measurements are independent, the variance of the fused estimate can be expressed as:

(4) $$\sigma_{\hat{x}}^{2}=\sum_{i=1}^{n}w_{i}^{2}\sigma_{i}^{2}$$

The fusion variance is minimized under the normalization constraint $\sum w_{i}=1$, and the Lagrangian function is constructed as follows:

(5) $$L=\sum_{i=1}^{n}w_{i}^{2}\sigma_{i}^{2}+\lambda \left(\sum_{i=1}^{n}w_{i}-1\right)$$

Set $\frac{\partial L}{\partial w_{i}}=0$ we obtain $2w_{i}\sigma_{i}^{2}+\lambda =0$, i.e., $w_{i}\propto \frac{1}{\sigma_{i}^{2}}$. Substituting the normalization constraint yields the optimal weight:

(6) $$w_{i}=\frac{1/\sigma_{i}^{2}}{\sum_{j=1}^{n}1/\sigma_{j}^{2}}$$

Under this condition, the variance after data fusion is minimized:

(7) $$\sigma_{\mathrm{wavg}}^{2}=\frac{1}{\sum_{i=1}^{n}1/\sigma_{i}^{2}}$$

### 2.3. Bias Estimation and Data Fusion Based on Kalman Filter

Kalman filtering is an optimal recursive estimation algorithm used to dynamically estimate system states from noisy observation data. Its core idea is to combine the system dynamic model with actual observation data through an iterative “prediction–update” procedure, thereby obtaining the optimal state estimate in a recursive manner. First, the current system state is predicted based on the state estimate from the previous time step and the system motion model. Then, the predicted state is corrected using the actual observation at the current time step. The correction weight is dynamically determined by the respective uncertainties of the prediction and the observation, where the information source with higher uncertainty is assigned a lower weight. This algorithm can efficiently fuse multi-source information in real time and gradually reduce the covariance of the estimation error under noisy and disturbed system conditions, thus achieving continuous, stable, and optimal estimation of system states. Therefore, it has been widely applied in navigation, control, signal processing, and other fields.

The state equation is expressed as:

(8) $$\begin{array}{l} y_{i}(t)=\omega (t)+b_{i}(t)+n_{i}(t)\\ {\dot{b}}_{i}(t)=w_{b_{i}}(t) \end{array}$$

where $y_{i}(t)$ denotes the angular velocity output of the gyroscope, ω(t) represents the true angular velocity, $n_{i}(t)$ stands for angle random walk (ARW), i.e., white noise; $b_{i}(t)$ is rate random walk (RRW), which can be expressed as noise driven by ω(t).

#### 2.3.1. Parameter Determination Based on Allan Variance

To ensure that the noise-covariance parameters in the Kalman filter are derived from the actual stochastic error characteristics of MEMS gyroscopes, rather than relying entirely on empirical tuning, this paper first performs Allan variance analysis on the static output data of each gyroscope.

On the log–log curve of Allan standard deviation, the segment with a slope of −1/2 is fitted to obtain the angle random-walk coefficient $N_{i}$, and the segment with a slope of +1/2 is fitted to obtain the rate random-walk coefficient $K_{i}$. The corresponding Allan variance expressions are given by

(9) $$\sigma_{\mathrm{ARW},i}^{2}(\tau )=\frac{N_{i}^{2}}{\tau }$$

(10) $$\sigma_{\mathrm{RRW},i}^{2}(\tau )=\frac{K_{i}^{2}\tau }{3}$$

where τ is the averaging time in Allan variance analysis.

For the stochastic error model adopted in Equation (8), $N_{i}^{2}$ and $K_{i}^{2}$ correspond to the continuous-time power spectral densities of the angular-velocity measurement white noise $n_{i}(t)$ and the bias-driving white noise $w_{b_{i}}(t)$, respectively, i.e.,

(11) $$E[n_{i}(t),n_{i}(t^{\prime })]=N_{i}^{2}\cdot \delta (t-t^{\prime })\;$$

(12) $$E[w_{b_{i}}(t),w_{b_{i}}(t^{\prime })]=K_{i}^{2}\cdot \delta (t-t^{\prime })\;$$

For a sampling interval τ, we adopt the following discrete-time noise variances. Therefore, the stochastic-error coefficients identified via Allan variance can establish a direct connection with the noise-covariance parameters in the Kalman filter. Among them, the measurement-noise covariance matrix of the original gyroscope array and the process-noise covariance matrix corresponding to bias random walk can be expressed as

(13) $$R_{i}=\frac{N_{i}^{2}}{\Delta t}$$

(14) $$Q_{\left\{b_{i}\right\}}=K_{i}^{2}\cdot \Delta t$$

It should be noted that static Allan variance analysis describes the random errors inherent in the gyroscope itself. Therefore, it can determine the zero-offset process noise $Q_{b}$ and the raw measurement noise $R_{\mathrm{raw}}$. However, it cannot determine the dynamic process noise of the external true angular velocity ω(t). The latter is only necessary in direct estimation models. Its value should be determined based on the dynamic range of the input angular velocity and the tracking performance required by the filter. Research has shown that the dynamic response of the direct estimation Kalman filter is closely related to the parameters of the true angular velocity process noise. These parameters should be set according to the dynamic characteristics of the input angular velocity. When the input signal changes slowly, using smaller process noise parameters helps reduce the filter’s effective bandwidth and improve noise suppression. As the input dynamics increase, these parameters need to be increased accordingly to enhance the filter’s ability to track rapid changes in angular velocity, thereby reducing amplitude attenuation and dynamic lag caused by insufficient filter bandwidth. Practical experiments also show that if the process noise parameters are too small, the filtering results may exhibit significant amplitude attenuation and phase lag.

Based on the above principle, $q_{\omega ,d}$ was selected according to the experimental condition. Under static conditions, bias stability and RMSE were used as the evaluation metrics, yielding $q_{\omega ,d}=3\times 10^{-3}{\left({}^{\circ}/s\right)}^{2}$. Under dynamic conditions, a larger value was required to improve the tracking capability of the filter, and the final horizontal displacement error of the inertial navigation solution was adopted as the system-level evaluation metric. Accordingly, $q_{\omega ,d}=3\times 10^{-1}{\left({}^{\circ}/s\right)}^{2}$ was selected.

Once the parameters are determined, they remain unchanged throughout all experiments under the corresponding operating conditions. The values mentioned above represent optimal settings determined based on the sensor’s noise level and the experimental conditions outlined in this paper. These values are intended to achieve a reasonable balance between noise suppression capabilities and dynamic tracking performance. They do not represent the globally optimal values for other sensors or under different operating conditions.

#### 2.3.2. Direct Estimation Kalman Filter

In the direct estimation model, the biases of all gyroscopes together with the common true angular velocity are simultaneously treated as system state quantities.

(15) $$X_{k}^{d}={\left[b_{\left\{1,k\right\}},b_{\left\{2,k\right\}},\ldots ,b_{\left\{N,k\right\}},\omega_{k}\right]}^{T}\;$$

Random-walk processes are adopted to describe the evolution of each state between adjacent sampling instants, and the process model is given as

(16) $$x_{k+1}^{d}=F_{d}\cdot x_{k}^{d}+w_{k}^{d},F_{d}=I_{N+1}\;$$

Its process-noise covariance matrix is given by

(17) $$Q_{d}=\left[\begin{array}{cc} Q_{b} & 0\\ 0^{T} & q_{\left\{\omega ,d\right\}} \end{array}\right]$$

where $Q_{b}$ corresponding to the biases is determined by the RRW coefficient obtained from Allan variance according to Equation (14), while $q_{\omega ,d}$ denotes the uncertainty in the discrete-time variation of the true input angular velocity, which varies under different operating conditions.

Organizing the measurement outputs of N gyroscopes into an observation vector, the measurement model can be established as

(18) $$Z_{k}^{d}=H_{d}\cdot X_{k}^{d}+V_{k}^{d}\;$$

(19) $$z_{k}^{d}={\left[y_{\left\{1,k\right\}},y_{\left\{2,k\right\}},\ldots ,y_{\left\{N,k\right\}}\right]}^{T}$$

the measurement matrix is given as

(20) $$H_{v}=\left[\begin{array}{ccccc} 1 & 0 & \ldots & 0 & 1\\ 0 & 1 & \ldots & 0 & 1\\ \vdots & \vdots & \ddots & \vdots & \vdots \\ 0 & 0 & \ldots & 1 & 1 \end{array}\right]$$

The measurement-noise covariance matrix adopts the original measurement-noise covariance obtained from Allan variance:

Therefore, in the direct estimation model, the physical quantities to be estimated, namely the individual gyroscope biases and the common angular velocity, are directly defined as system states, while the actual angular-velocity outputs measured by the gyroscope array serve as observations.

From the perspective of the measurement matrix H, the static Kalman filter takes the common angular velocity and each bias term b as state variables. However, due to

(21) rank(H)=n

However, since the dimension of our state vector is n+1, the direct estimation model is not fully observable for all states.

To solve for the unobservable mode, define the unobservable vector $\eta ={\left[\begin{array}{c} \eta_{1},\ldots ,\eta_{N},\eta_{\omega } \end{array}\right]}^{T}$. The solution yields $\eta_{1}=\eta_{2}=\cdots =\eta_{N}=-\eta_{\omega }$. The unobservable subspace is spanned by the vector $\eta_{0}={\left[\begin{array}{c} -1,-1,\ldots ,-1,1 \end{array}\right]}^{T}\in {\mathbb{R}}^{N+1}$. Physically, this unobservable mode corresponds to the fact that the sum of the common component of rate random walk of all gyros and the true angular rate cannot be distinguished by measurements.

However, although the measurement equation itself cannot uniquely separate $\omega_{k}$ and the common bias, the process noise covariance matrix $Q=\mathrm{diag}\left(q_{\omega },q_{b_{1}},\ldots ,q_{b_{N}}\right)$ provides statistical constraints for the filter. The unobservable common mode is constrained by the initial conditions, prior covariance and process noise model, while the noise and relative drift in the observable subspace are suppressed by the redundant information of the sensor array.

#### 2.3.3. Indirect Estimation Kalman Filter

The indirect estimation Kalman filter constructs differential observations among gyroscopes to eliminate the common true angular rate and estimate the relative biases. The state vector is defined as

(22) $$x_{k}^{i}={\left[b_{\left\{1,k\right\}},b_{\left\{2,k\right\}},\ldots ,b_{\left\{N,k\right\}}\right]}^{T}\;$$

According to the random-walk model for gyroscope biases, its process model is given by

(23) $$x_{k+1}^{i}=x_{k}^{i}+w_{k}^{i}\;$$

where the process-noise covariance is $Q_{i}=Q_{b}\;$ which is determined from the RRW coefficients obtained by Allan variance analysis.

According to the cyclic-difference structure adopted in this paper, the difference matrix is defined as

(24) $$D=\left[\begin{array}{ccccc} -1 & 1 & 0 & \ldots & 0\\ 0 & -1 & 1 & \ldots & 0\\ \vdots & \vdots & \ddots & \ddots & \vdots \\ 0 & 0 & \ldots & -1 & 1\\ 1 & 0 & \ldots & 0 & -1 \end{array}\right]$$

Applying this difference matrix to the original gyroscope measurement vector, the common true angular velocity shared by all gyroscopes can be completely eliminated:

(25) $$z_{k}^{i}\;=D\cdot y_{k}=D\cdot \left(\omega_{k}\cdot l_{N}+b_{k}+n_{k}\right)=D\cdot b_{k}+D\cdot n_{k}$$

Therefore, the differential measurement model can be expressed as:

(26) $$z_{k}^{i}=H_{i}\cdot x_{k}^{i}+v_{k}^{i},H_{i}=D$$

where

(27) $$v_{k}^{i}=D\cdot n_{k}$$

The corresponding actual differential observation is given by

(28) $$z_{k}^{i}={\left[y_{\left\{2,k\right\}}-y_{\left\{1,k\right\}},y_{\left\{3,k\right\}}-y_{\left\{2,k\right\}},\ldots ,y_{\left\{1,k\right\}}-y_{\left\{N,k\right\}}\right]}^{T}$$

Thus, the differential observations contain only the relative biases and differential measurement noise, while the common true angular rate is eliminated.

Since the differencing operation changes the measurement-noise characteristics, the original measurement-noise covariance cannot be used directly. Instead, the differential measurement-noise covariance is obtained by covariance propagation:

(29) $$R_{\mathrm{diff}}=D\cdot R_{\mathrm{raw}}\cdot D^{T}\;$$

Although the original gyroscope measurement noises may be mutually independent, the differential noises are generally correlated because adjacent differential observations may share the noise of the same gyroscope. Therefore, $R_{\mathrm{raw}}$ accounts for the correlation introduced by the differencing operation.

There still exists an unobservable common-bias mode in the differential model, because:

(30) rank(H)=n−1<n

To solve for the unobservable mode, solving the equation HX=0 yields the following system of equations:

(31) $$\left\{\begin{array}{l} -X_{1}+X_{2}=0,\\ -X_{2}+X_{3}=0,\\ \vdots \\ -X_{N-1}+X_{N}=0,\\ X_{1}-X_{N}=0 \end{array}\right.\to X_{1}=X_{2}=\ldots =X_{N}$$

Indirect operation attenuates the relative drift among gyros and suppresses random noise via the compensated average output. The final angular rate estimate is generally expressed as:

(32) $${\hat{\omega }}_{k}=\frac{1}{N}\sum_{i=1}^{N}\left(y_{i,k}-{\hat{b}}_{i,k}\right)$$

Substituting $y_{i,k}=\omega_{k}+b_{i,k}+n_{i,k}$ yields

(33) $${\hat{\omega }}_{k}-\omega_{k}=\frac{1}{N}\sum_{i=1}^{N}\left(b_{i,k}-{\hat{b}}_{i,k}\right)+\frac{1}{N}\sum_{i=1}^{N}n_{i,k}$$

If the indirect filter can accurately estimate the relative bias, i.e.,

(34) $$b_{i,k}-{\hat{b}}_{i,k}\approx b_{c,k}$$

the dominant estimation error becomes

(35) $${\hat{\omega }}_{k}-\omega_{k}\approx b_{c,k}+{\bar{n}}_{k}$$

where

(36) $${\bar{n}}_{k}=\frac{1}{N}\sum_{i=1}^{N}n_{i,k}$$

Therefore, the indirect estimation Kalman filter estimates and compensates for the relative bias differences among the gyroscopes, while subsequent averaging further suppresses uncorrelated measurement noise. However, without an external absolute angular-rate reference, the common bias component remains unobservable.

The ARW coefficients are used to construct the original measurement-noise covariance $R_{\mathrm{raw}}$, whereas the RRW coefficients determine the bias process-noise covariance $Q_{b}$. Unlike the direct estimation model, the indirect estimation model eliminates the common angular rate through differential measurements and therefore does not require an additional process-noise parameter for the true angular rate.

Although the models are not fully observable, this does not prevent their application to virtual gyroscope fusion. The objective is to obtain a low-noise, low-drift angular-rate estimate rather than to uniquely reconstruct all internal states. Therefore, partial unobservability mainly limits the uniqueness of state interpretation rather than the effectiveness of the fused angular-rate estimation.

## 3. Analysis of the Improvement in Bias Stability Through Data Fusion

### 3.1. Experimental Setup

**Figure 1:**
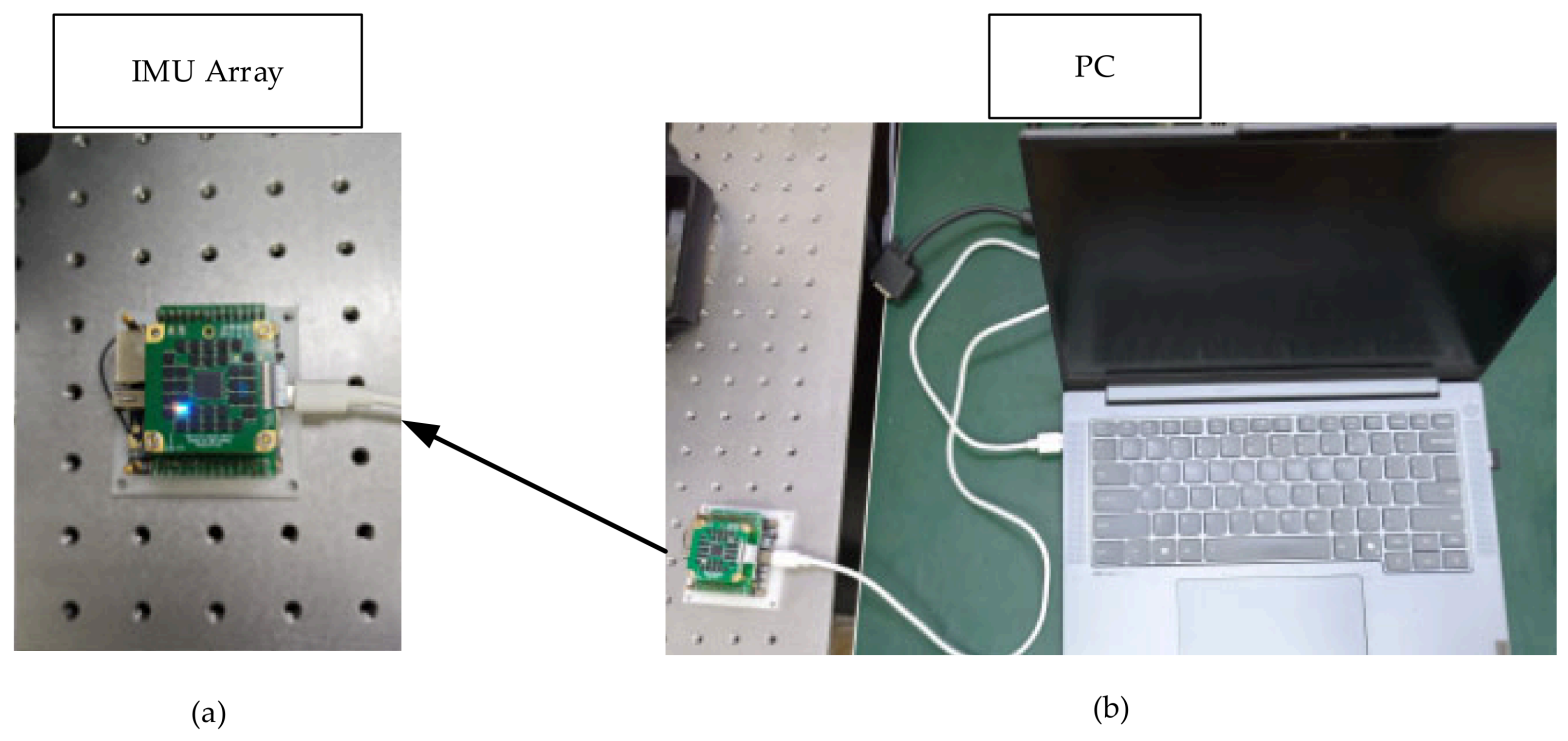
Experimental hardware system: (**a**) MEMS IMU array circuit board; (**b**) static data acquisition experimental setup.

To conduct the physical experiments, an experimental platform for testing the IMU array was established. As shown in Figure 1 in the experimental setup, the object under test was a highly integrated 32-unit IMU array module, which was fixed on a white load plate. The module integrates multiple IMU sensor chips, peripheral interface circuits, and operating status indicators, enabling the synchronous acquisition of multi-channel inertial information. The IMU array module was connected to an external data acquisition system through several test cables, which provided both power supply and data communication functions, thereby enabling the real-time transmission of inertial measurement data, including angular velocity and acceleration.

The entire IMU array testing device was mounted on an optical vibration isolation platform with standard threaded holes. This platform can effectively reduce the influence of environmental vibration on the experimental results and provide stable mechanical support during the test, thereby ensuring the stability of data acquisition and the reliability of the measurement results.

The system adopts the RV1106G3 main control chip paired with an ICE40UP5K field-programmable gate array (FPGA) to acquire array data in parallel at a frequency of 200 Hz. The experimental data in this paper are collected by a multiple inertial measurement unit (MIMU) array consisting of 32 ICM42688-P micro-electro-mechanical inertial measurement units (MEMS IMUs), with a sampling duration of 8 h. During the experiment, the IMU array was placed horizontally in a static state, and the angular velocity and acceleration data along the x-, y-, and z-axes of each IMU unit in the array were synchronously collected. After data acquisition, the raw experimental data were subjected to integrity checking, outlier screening, and accuracy verification to ensure their validity and reliability. These processed data provide a reliable basis for subsequent analysis of data fusion algorithms and performance evaluation.

### 3.2. Simulation and Experiments on the Improvement of Bias Stability via Data Fusion

The simulation experimental model is given as follows:

The array consists of N simulated gyroscopes. Each gyroscope undergoes R independent runs, and each run contains K sampling points. In the *r*-th run, the simulated output of the *i*-th gyroscope at the *k*-th sampling instant is expressed as:

(37) $$s_{i}^{\left(r\right)}[k]=u_{\mathrm{ref}}[k]+\varepsilon_{i}^{\left(r\right)}[k]$$

The white noise is generated as follows:

(38) $$\varepsilon_{i}^{\left(r\right)}[k]=\sigma_{\varepsilon ,i}\xi_{i}^{\left(r\right)}[k]$$

where

(39) $$\xi_{i}^{\left(r\right)}[k]\sim N(0,1)$$

Therefore,

(40) $$\varepsilon_{i}^{\left(r\right)}[k]\sim N\left(0,\sigma_{\varepsilon ,i}^{2}\right)$$

which satisfies

(41) $$E\left[\varepsilon_{i}^{\left(r\right)}[k]\right]=0$$

(42) $$\mathrm{Var}\left[\varepsilon_{i}^{\left(r\right)}[k]\right]=\sigma_{\varepsilon ,i}^{2}$$

Assume that the white noises corresponding to different gyroscopes, different runs and different sampling instants are mutually independent, then we have

(43) $$E\left[\varepsilon_{i}^{\left(r\right)}[k]\varepsilon_{p}^{\left(q\right)}[l]\right]=0,(i,r,k)\neq (p,q,l)$$

where $y_{i}^{\left(j\right)}(k)$ denotes the simulated output of the i-th gyroscope at the k-th sampling instant during the j-th independent run; $\omega_{\mathrm{true}}(k)$ is the prescribed true angular rate at the k-th sampling instant; $n_{i}^{\left(j\right)}(k)$ is the Gaussian white noise of the i-th gyroscope during the j-th run; $\sigma_{n,i}$ is the prescribed white-noise standard deviation of the i-th gyroscope; and $\xi_{i}^{\left(j\right)}(k)$ is an independent random variable following the standard normal distribution N(0,1). Here, i=1,2,…,N denotes the gyroscope index, j=1,2,…,J denotes the independent-run or power-on-cycle index, and k=1,2,…,K denotes the sampling-point index within a single run. The white-noise samples generated for different gyroscopes, runs, and sampling instants are assumed to be mutually independent; therefore, their cross-correlation expectation is zero when (i,j,k)≠(p,q,l).

Two groups of simulation experiments are designed, and the simulation parameters are shown in Table 1:

The first group of experiments adopts IMUs with identical standard deviation for simulation, while the second group uses IMUs with varying standard deviation. The simulation results are listed in the following table:

The simulation results in Table 2 show that when all IMUs share identical standard deviation, the fused standard deviation obtained by the numerical averaging method, weighted least squares method and indirect estimation Kalman filter is basically consistent. When the IMUs have inconsistent standard deviation, the weighted least squares algorithm assigns higher weights to IMUs with superior standard deviation and lower weights to those with poor standard deviation via the weight formula, thus achieving better fused standard deviation compared with the numerical averaging method.

**Figure 2:**
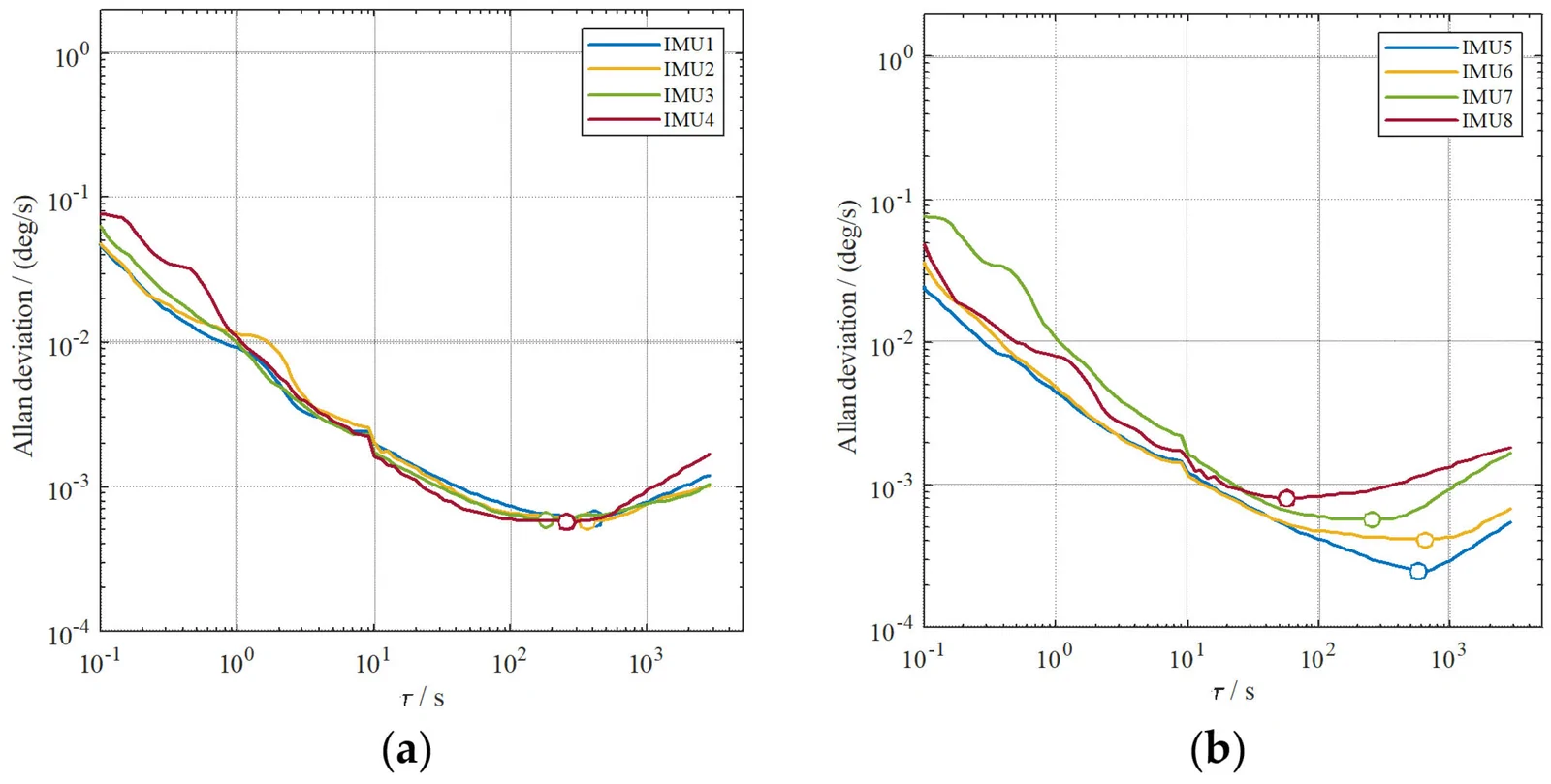
Allan deviation curves of IMU1–IMU8: (**a**) Allan deviation curves of IMU1–IMU4; (**b**) Allan deviation curves of IMU5–IMU8.

Subsequently, physical test data are employed for experimental validation. Allan variance analysis is conducted on the measured dataset to calculate the bias stability. The single-axis Allan curves of the eight IMUs from the two test groups are presented in Figure 2.

Bias-stability parameters of IMU 1–IMU 8 are obtained from Allan-curve analysis. The IMUs in the first test group possess similar bias stability, while those in the second test group exhibit considerable differences in bias stability. The detailed bias-stability results calculated via Allan-variance analysis are listed in Table 3.

**Figure 3:**
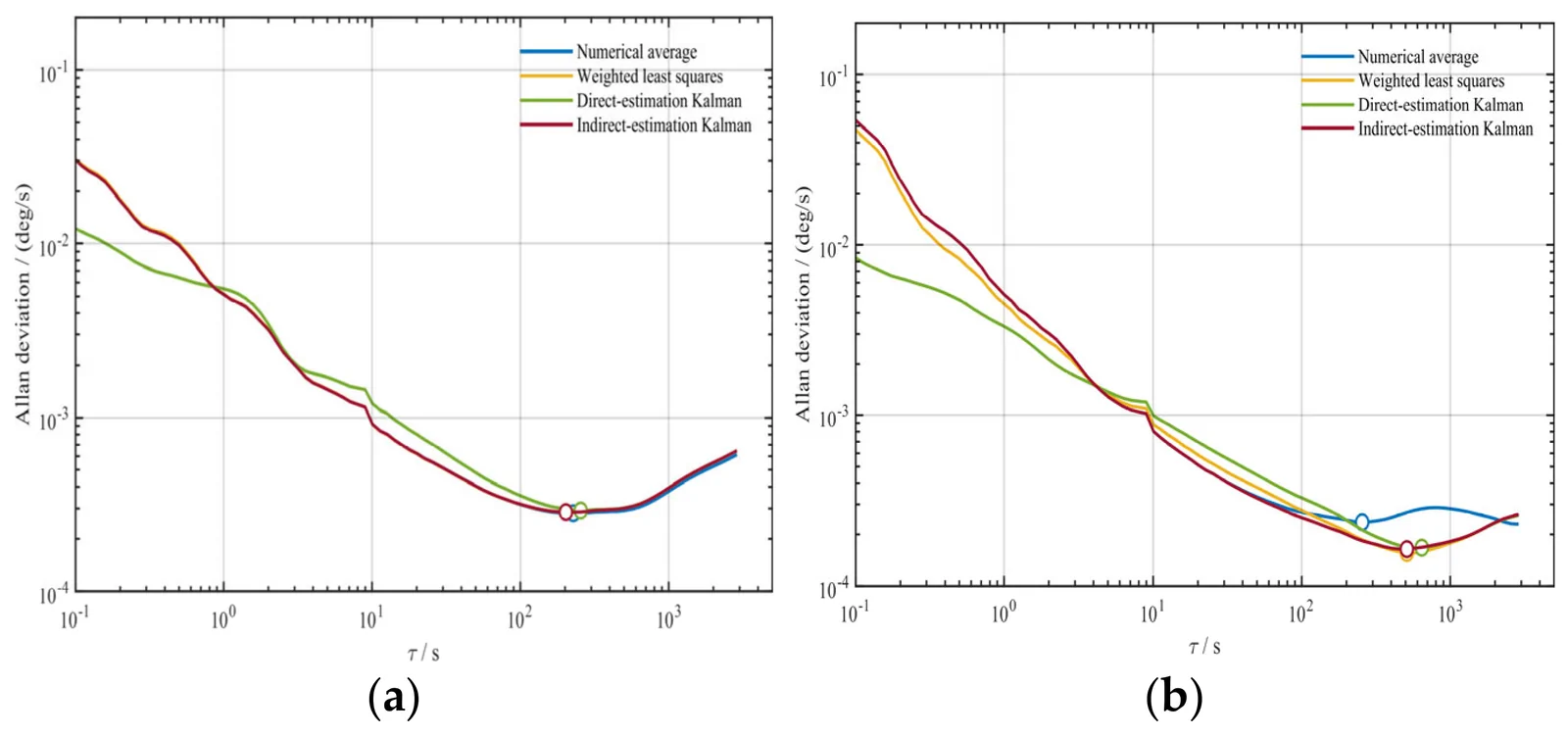
Allan curves of the four fusion methods: (**a**) Allan deviation curves of four fusion methods in Experiment 1. (**b**) Allan deviation curves of four fusion methods in Experiment 2.

Subsequently, the numerical averaging method, weighted least squares method, direct estimation Kalman filter and indirect estimation Kalman filter are adopted for data fusion. Allan variance analysis is performed on the fused data to acquire the characteristic parameters of the fused IMU signals, and the corresponding Allan curves are shown as Figure 3:

As shown in Table 4, the bias stability values for different fusion methods in the two groups of experiments are extracted from the Allan curves of fused data. In Experiment 1, the four IMUs exhibit nearly identical bias stability, and all fusion algorithms degrade to the numerical averaging method, thus yielding consistent bias stability results. In Experiment 2, the IMUs have distinct bias stability performance. The weighted least squares method achieves superior performance by adaptive weight adjustment. Meanwhile, the direct estimation Kalman filter and indirect estimation Kalman filter also outperform simple numerical averaging via iterative state prediction and measurement update.

### 3.3. Analysis of Computational Efficiency of Data Fusion Algorithm

In addition to accuracy metrics, the computational efficiency of algorithms is an essential factor that must be considered for the engineering application of IMU arrays. The numerical averaging method and weighted least squares method are essentially algebraic fusion algorithms. A single fusion cycle mainly involves addition, multiplication and normalization operations, which generally consumes little computation time in practice. In contrast, Kalman filtering algorithms require the maintenance of state vectors, covariance matrices and gain matrices. As the number of IMUs participating in fusion rises, the state dimension and the scale of matrix operations increase simultaneously, resulting in a more remarkable growth in computational latency.

In this paper, measured data from the same batch of the 32-unit IMU hardware shown in the above figure are adopted to conduct computation time comparisons. We add 3 IMUs into the fusion system at each step, and record the running time of the four algorithms when the number of IMUs ranges from 3 to 30. This experiment can reveal the differences in real-time performance of various algorithms as the array scale expands, and provide a reference for the subsequent selection of array size and practical engineering deployment. To intuitively observe how the number of IMUs affects computational overhead, the fusion test is carried out by incrementing 3 IMUs each time. The horizontal axis represents the number of IMUs involved in data fusion (3 to 30), while the vertical axis denotes the corresponding computation time of each algorithm.

**Figure 4:**
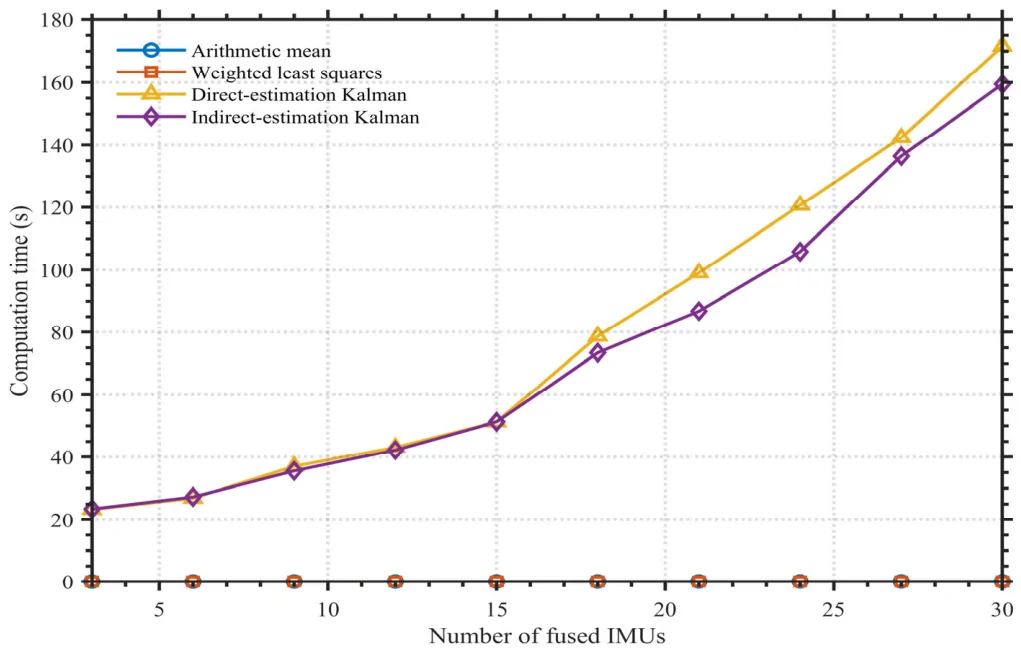
Comparison of time consumption for four methods.

As shown in Figure 4, experimental runtime comparison demonstrates that numerical averaging and weighted least squares maintain stable, low latency regardless of IMU quantity. However, both direct and indirect estimation Kalman filters suffer rapidly rising computational overhead with more IMUs. Their efficiency degrades severely, with runtime exhibiting nearly exponential growth against the number of fused IMUs.

## 4. Analysis of the Influence of Data Fusion on Measurement Accuracy

The second simulation experiment introduces power-on repeatability on the basis of the first simulation. Its model is given as follows:

(44) $$y_{i}^{\left(j\right)}(k)=\omega_{\mathrm{true}}(k)+b_{i}^{\left(j\right)}+n_{i}^{\left(j\right)}(k)$$

The power-on repeatability error is defined as

(45) $$b_{i}^{\left(j\right)}=\sigma_{r,i}\zeta_{i}^{\left(j\right)}$$

where

(46) $$\zeta_{i}^{\left(j\right)}\sim N(0,1)$$

Therefore,

(47) $$b_{i}^{\left(j\right)}\sim N\left(0,\sigma_{r,i}^{2}\right)$$

Its statistical properties are given by

(48) $$E\left[b_{i}^{\left(j\right)}\right]\equiv 0\;\mathrm{Var}\left[b_{i}^{\left(j\right)}\right]=\sigma_{r,i}^{2}$$

Here, $b_{i}^{\left(j\right)}$ remains constant for all K sampling points in the *j*-th run, but is randomly regenerated across different runs:

(49) $$b_{i}^{\left(j\right)}(1)=b_{i}^{\left(j\right)}(2)=\ldots =b_{i}^{\left(j\right)}(K)$$

Meanwhile, it is assumed that the power-on repeatability error and white noise are mutually independent:

(50) $$E\left[b_{i}^{\left(j\right)}n_{p}^{\left(q\right)}(k)\right]=0$$

where $b_{i}^{\left(j\right)}$ denotes the repeatability bias of the *i*-th gyroscope generated after the *j*-th run or power-up; $\left(\sigma_{r,i}\right)$ is the preset power-on repeatability standard deviation of the *i*-th gyroscope; $\zeta_{i}^{\left(j\right)}$ represents the standard normal random variable used to generate the power-on repeatability error. Among them, the white noise model is consistent with the simulation model in the previous chapter.

In Simulation 1, all gyroscopes share identical power-on repeatability values, while their standard deviation values increase sequentially.

In Simulation 2, the standard deviation values of all gyroscopes rise progressively, whereas their repeatability values decline progressively.

The parameter configurations of the two simulation cases are listed in Table 5.

Monte-Carlo simulations are performed to assess fusion performance and guarantee statistically valid results. Each group contains $N_{MC}=1000$ independent trials, with 10 power-up cycles conducted per trial. The root mean square error (RMSE) of every fusion strategy is computed across all power-up periods.

(51) $$\mathrm{RMSE}=\sqrt{\frac{1}{N}\sum_{k=1}^{N}{\left(\hat{\omega }(k)-\omega (k)\right)}^{2}}$$

To analyze the influence of different error characteristics on the fusion performance of multiple gyroscopes, two groups of simulation experiments are designed in this paper. In the experiments, the standard deviation and power-on repeatability parameters of gyroscopes are modified separately, and the performance differences among several fusion methods are compared. The fusion accuracy of each algorithm is evaluated by the root mean square error (RMSE) in units of deg/h, and this evaluation metric covers the improvement effects on both standard deviation and power-on repeatability. The RMSE characterizes the overall deviation of fused outputs under static zero-input conditions; for simulation tests where the true angular velocity is predefined by the model, RMSE directly quantifies the error between fused estimates and ground-truth values. Unlike standard deviation, RMSE is jointly affected by power-on repeatability, constant offset, random noise and low-frequency drift, making it more suitable for evaluating the comprehensive measurement accuracy of fusion results.

In this chapter, standard deviation and RMSE are analyzed as two complementary indicators: the former characterizes the random fluctuation of gyroscope output sequences and only reflects the dispersion of measurements around their own mean value; the latter focuses on the overall accuracy of fused outputs with respect to ground truth under static measurement scenarios, covering both random fluctuations and fixed systematic biases. If an algorithm achieves favorable standard deviation yet exhibits deteriorated RMSE, it indicates that its weight design only optimizes the single metric of random fluctuation without simultaneously mitigating fixed errors or power-on repeatability errors.

The weight expression of the numerical averaging method is given as:

(52) $$W_{AG}=\mathrm{diag}\left(1\;1\;1\;1\right)$$

The weights of the weighted least squares method are related to noise variance according to its formula. The weights of the weighted least squares method in Simulation 1 are expressed as follows:

(53) $$W_{Weighted}=\mathrm{diag}{\left(0.1^{2},0.2^{2},0.3^{2},0.4^{2}\right)}^{-1}$$

In contrast, the direct estimation Kalman filter and indirect estimation Kalman filter assign different weights to IMU predicted values and measured true values based on model parameters and Kalman gain K through continuous recursion and update, and then output the estimated true angular velocity.

The fusion accuracy of the four algorithms for the RIMU array in Monte-Carlo simulations is presented in Table 6.

The data fusion accuracy under the above-mentioned two simulation cases is presented in Table 6. In Simulation 1, all gyroscopes share identical power-on repeatability error parameters, and the weights of the weighted least-squares method are constructed solely based on the preset standard deviation of each gyroscope. Consequently, the main discrepancy among different gyroscopes originates from standard deviation. The simulation results show that the weighted least-squares method and direct estimation Kalman filtering achieve smaller standard deviation and root-mean-square error (RMSE) compared with the numerical averaging method and indirect estimation Kalman filtering.

In Simulation 2, the standard deviation of each gyroscope increases progressively, while the power-on repeatability error parameters decrease gradually, which means the two types of error characteristics exhibit opposite variation trends. The weighted least-squares method constructs fixed weights merely according to standard deviation. As a result, the IMU with the minimum standard deviation is assigned the largest weight, whereas this IMU suffers from relatively large power-on repeatability error. The results demonstrate that the weighted least-squares method reduces the fused standard deviation to 0.08 °/h, better than 0.14 °/h obtained by numerical averaging. Nevertheless, its RMSE rises to 0.29 °/h, which is higher than the 0.19 °/h of numerical averaging. This reveals that when different error components show conflicting variation trends, constructing fixed weights based only on a single statistical indicator may lead to weight mismatch. Summarizing the two groups of simulation results, under the static model, prior settings and parameter conditions adopted in this paper, direct estimation Kalman filtering yields the minimum standard deviation and RMSE in both cases. By comparison, for the weighted least-squares method in Simulation 2, although the standard deviation is improved by weight assignment purely from standard deviation, the overall RMSE deteriorates instead.

We conduct experimental analysis on single-axis data of each IMU using the IMU measurement data collected as shown in the above figure, and calculate bias stability via Allan variance analysis. The bias stability values of the eight IMUs divided into two groups are listed in Table 7. Afterwards, the IMUs are split into two groups, and data fusion experiments are performed with the aforementioned fusion algorithms.

The weight formula of the numerical averaging method for Group 1 is given as follows:

(54) $$W_{AG}=\mathrm{diag}\left(1\;1\;1\;1\right)$$

The weight expression for the weighted least squares method is as follows:

(55) $$W_{Weighted}=\mathrm{diag}{\left(3.26^{2},3.13^{2},3.20^{2},3.09^{2}\right)}^{-1}$$

Next comes the experimental analysis of Group 2.

The weight formula of the numerical averaging method is given as follows:

(56) $$W_{AG}=\mathrm{diag}\left(1\;1\;1\;1\right)$$

The weight expression for the weighted least squares method is as follows:

(57) $$W_{Weighted}=\mathrm{diag}{\left(1.35^{2},2.22^{2},3.09^{2},4.35^{2}\right)}^{-1}$$

As shown in Table 8, the experimental results reveal the above-mentioned phenomenon. In terms of the fused bias stability of Group 1, the four fusion methods deliver nearly identical improvements, which indicates that when all IMUs possess similar bias stability, both the Kalman filter and weighted least squares degrade to simple averaging. In terms of the fused RMSE, the numerical averaging method, weighted least squares method and dynamic Kalman filter achieve comparable fusion performance, while the static Kalman filter yields the optimal results with static constraints introduced.

As for the fused bias stability of Group 2, only numerical averaging exhibits slightly inferior performance, and the other three fusion methods realize almost the same degree of improvement in bias stability. When evaluating the fused RMSE, the numerical averaging method and dynamic Kalman filter perform similarly, the weighted least squares method produces the worst results, and the static Kalman filter still achieves the best performance under the given static constraints.

## 5. Dynamic Experimental Validation

**Figure 5:**
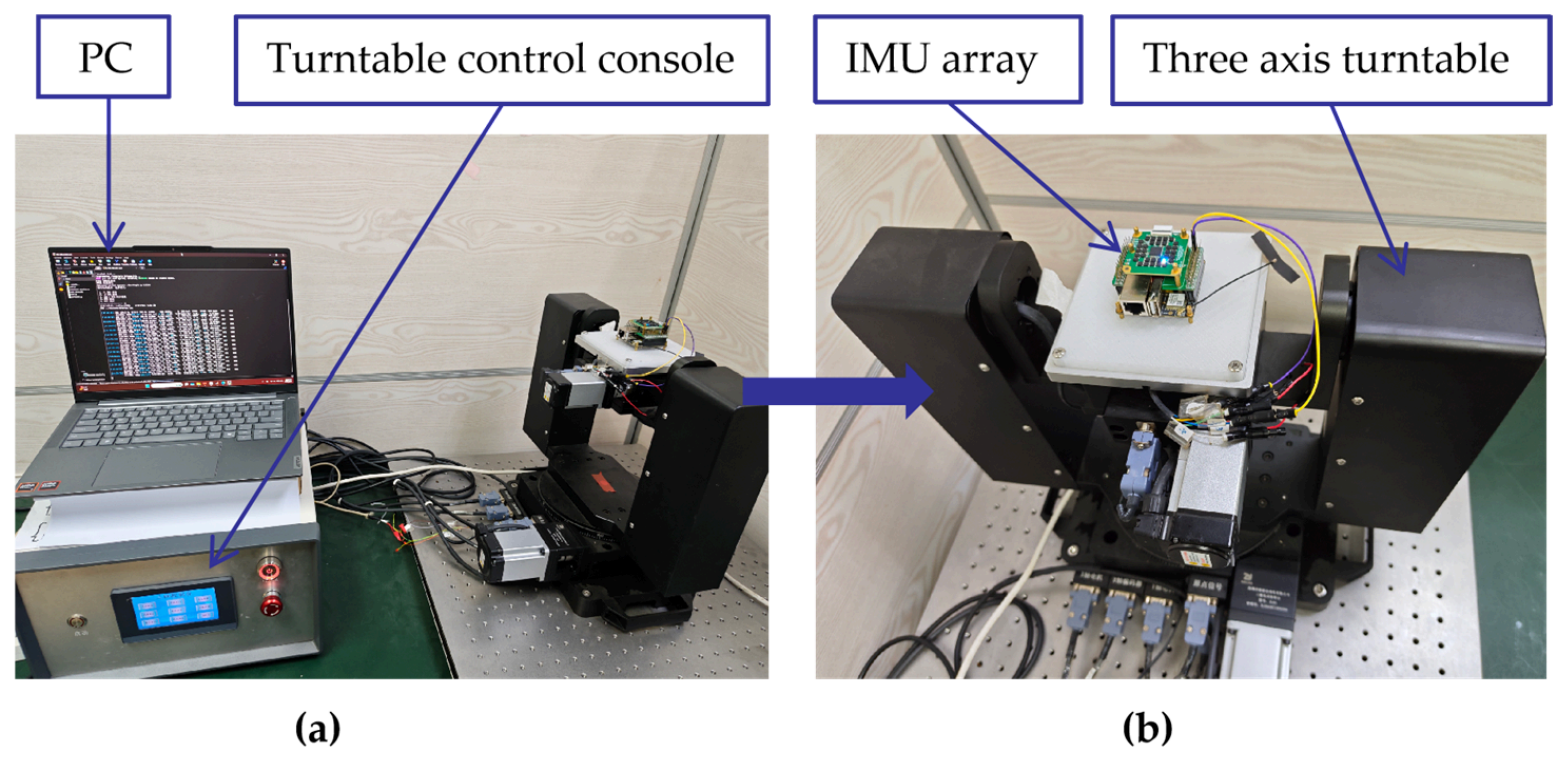
Dynamic turntable experimental setup. (**a**) Overall view of the experimental setup. (**b**) IMU array mounted on the three-axis turntable.

The dynamic experimental platform is shown in Figure 5. During the experiment, the IMU array was rigidly mounted on a three-axis turntable, and the turntable was installed on a vibration-isolation platform to reduce the influence of external vibration on the test results. After the IMU array and data acquisition system were connected, the turntable control system was powered on, and the motion sequence, rotation axis, angular velocity, and duration of each stage were configured through the turntable controller. During the experiment, the turntable executed the prescribed three-axis motion sequence, while the host computer continuously and synchronously acquired the three-axis angular velocity and three-axis acceleration data from the four IMUs. The data acquisition program on the PC was used to monitor the sensor outputs and communication status in real time, thereby ensuring the continuity and validity of the acquired data.

After the system connection and operating status were verified, the dynamic turntable experiment was started. The IMU array was first kept stationary for 10 min, followed by constant-angular-rate rotations about the z-, x-, and y-axes in sequence. The angular rate for each rotation stage was set to $30^{\circ }/s$, with a duration of 80 s. A 20 s stationary interval was inserted between successive rotation stages, and another 20 s stationary period was maintained after the final rotation. The initial stationary data were used for bias estimation, noise parameter analysis, and initial alignment, while the subsequent approximately 300 s of data were used to evaluate the dynamic fusion and inertial navigation performance.

The initial 10 min stationary data were mainly used for calibration, parameter estimation, and initial alignment and were excluded from the subsequent navigation evaluation. Inertial navigation started from the beginning of the formal Z-axis rotation, resulting in an effective navigation duration of approximately 300 s. For each fusion method, the corresponding measurements from the four IMUs were fused independently along each axis. Specifically, the four x-, y-, and *z*-axis gyroscope outputs and the corresponding x-, y-, and *z*-axis accelerometer outputs were processed using the same fusion method, resulting in three-axis fused angular-velocity and three-axis fused specific-force measurements. The resulting six-axis fused measurements were then used as the inputs to the PSINS inertial navigation system for continuous attitude, velocity, and position estimation. To ensure a fair comparison, the same raw dataset, navigation interval, initial alignment conditions, and PSINS configuration were used for all four methods, with the fusion algorithm being the only difference among the four navigation solutions. Since the turntable remained fixed in position throughout the experiment, the theoretical horizontal displacement was zero. Therefore, the final horizontal position drift at the end of navigation was selected as the primary performance metric for the dynamic experiment.

**Figure 6:**
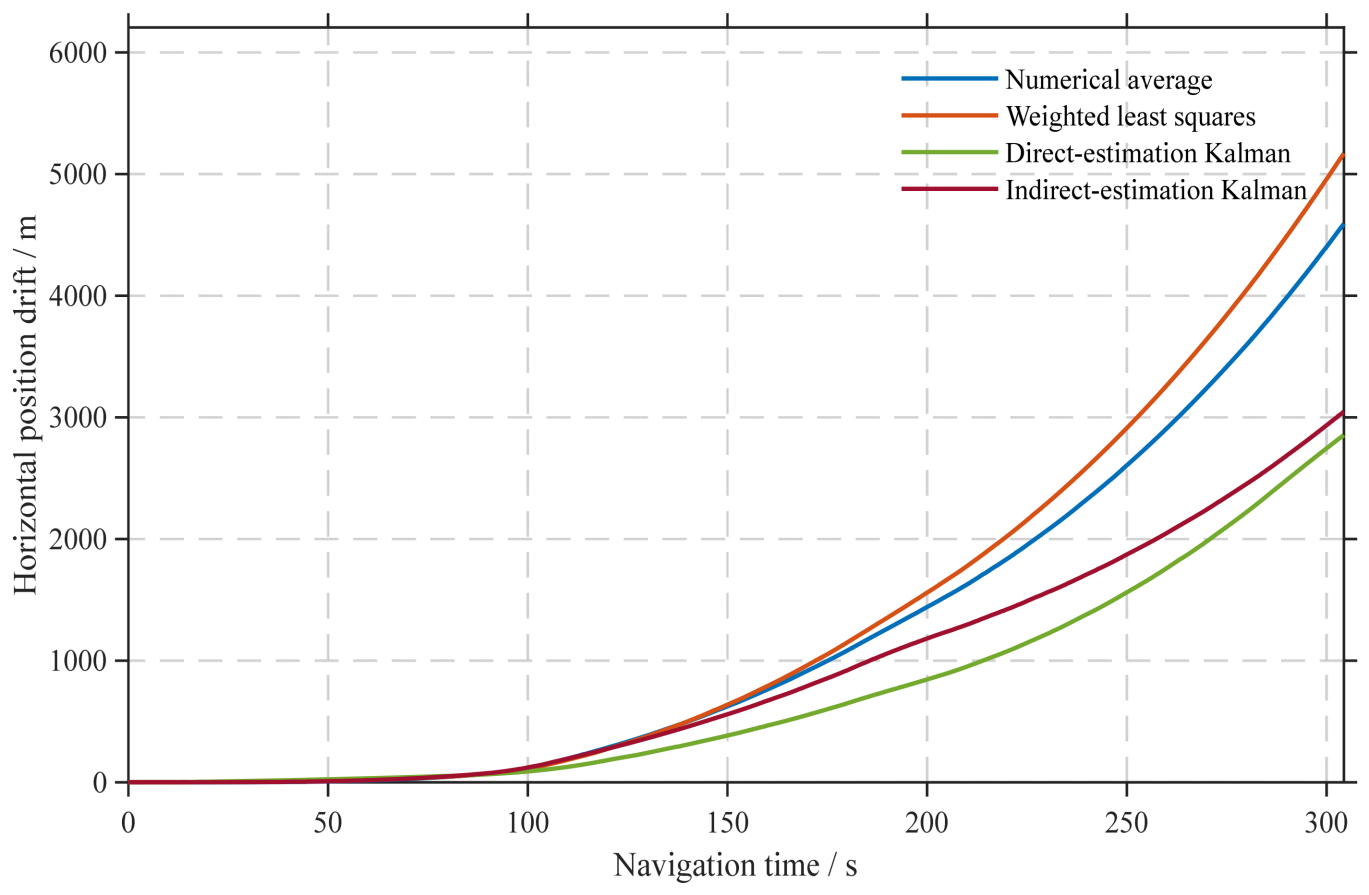
Horizontal position drift of the four fusion methods during the dynamic navigation experiment.

As shown in Figure 6, the experimental results show clear performance differences among the four fusion methods under dynamic navigation conditions. The direct estimation Kalman filter achieved the smallest final horizontal position drift of 2855 m and therefore exhibited the best performance. The indirect estimation Kalman filter ranked second, with a final horizontal position drift of 3048 m. By comparison, the numerical averaging method and weighted least squares method produced final horizontal position drifts of 4593 m and 5171 m, respectively, both substantially larger than those obtained using the two Kalman-filter-based methods. In particular, the fixed weighting strategy did not maintain an advantage under the more complex dynamic conditions and even performed worse than the simple averaging method.

## 6. Investigation on the Effect of IMU Array Scale Variation on Performance Improvement

To investigate the effect of varying IMU quantities on bias stability, a simulation with 96 IMUs featuring nearly identical bias stability parameters is conducted in this work. For multi-IMU data fusion scenarios where all IMUs have identical bias stability parameters, different fusion algorithms produce nearly equivalent final fusion performance. In this case, both the weighted least squares fusion algorithm and Kalman filter lose their unique characteristics and gradually degenerate into straightforward numerical averaging. Under this fusion mechanism, the overall system accuracy after fusion improves steadily as the number of participating IMUs increases, and the magnitude of accuracy enhancement strictly follows the classical inverse square-root-of-n law.

**Figure 7:**
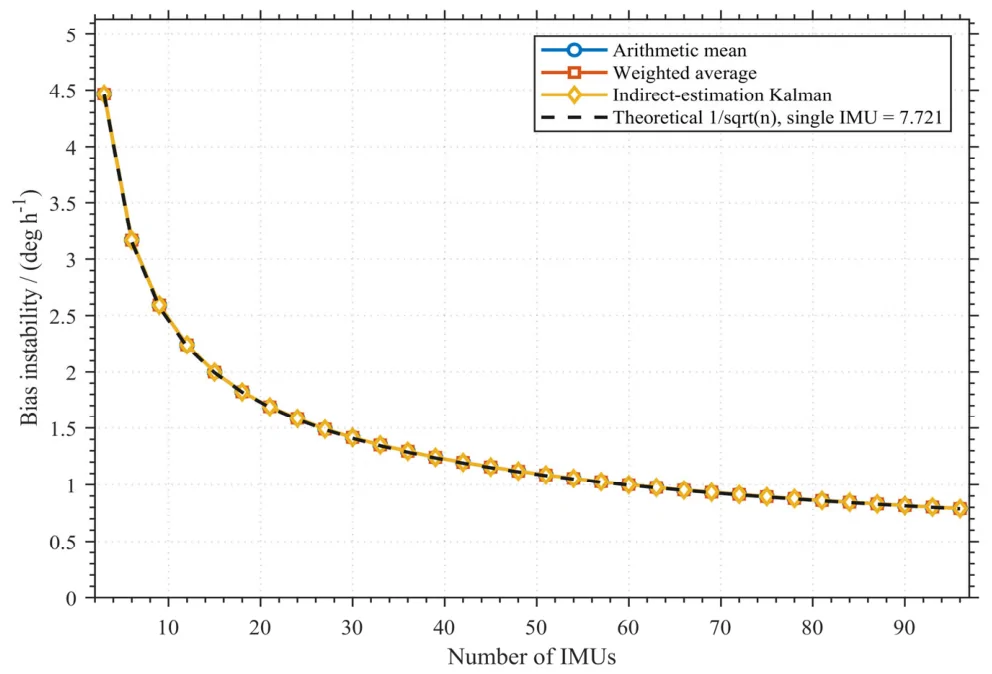
Variation in bias stability with the number of IMUs under four fusion methods.

The results are shown in Figure 7.

In multi-IMU fusion systems with mutually independent and comparable sensor errors, bias stability generally decreases approximately according to the $1/\sqrt{N}$ law as the number of IMUs increases. A rise in the number of IMUs leads to linear growth in hardware cost, power consumption, volume, and computational complexity. Furthermore, practical engineering applications are constrained by inherent residual systematic errors of IMUs, noise correlation, and environmental disturbances, which impose a lower bound on achievable accuracy. Therefore, a reasonable trade-off between accuracy improvement and engineering cost should be identified. This paper adopts a marginal benefit analysis method to calculate the absolute and relative improvements in bias stability brought by adding a fixed number of IMUs, locate the inflection point where accuracy D thereby identify a reasonable IMU array size with favorable cost-effectiveness.

To determine a reasonable IMU array-size range, two complementary criteria are adopted in this paper based on the performance curve STD(*n*) with bias stability as the evaluation metric:

(1) Marginal Improvement Criterion

(58) $$\begin{array}{l} \Delta (n)=\mathrm{STD}(n-3)-\mathrm{STD}(n)\\ \eta (n)=\frac{\mathrm{STD}(n-3)}{\Delta (n)}\times 100\% \end{array}$$

The system is considered to enter the interval of diminishing marginal returns when the following condition is satisfied:

η(n)<5%or Δ(n)<0.05deg/h

(2) Automatic Knee Point Detection

After normalizing the STD(*n*) curve, calculate the perpendicular distance from each data point to the line connecting the first and last points, and take the point with the maximum distance as the geometric knee point.

(59) $$n_{\mathrm{opt}}=\arg\max d(n)$$

This point marks the transition where performance improvement shifts from a “rapid decline phase” to a “slow decline phase”.

As illustrated in the above figure, the system bias stability exhibits typical non-linearly decaying optimization characteristics with the increase in the number of deployed IMUs, and the overall performance improvement trend varies across stages. The measured curve shows that when the IMU count rises from 3 to 30, the bias stability rapidly improves from 4.46 °/h to 1.42 °/h, representing a reduction of 68.2%. When the count further increases from 30 to 60, the index drops to 1.00 °/h with a markedly slowed performance gain rate, where the bias stability only decreases by 0.42 °/h in this stage. Once the quantity exceeds 60, the indicator fluctuates within a narrow range of 1.00–0.79 °/h. The absolute improvement between adjacent intervals is generally less than 0.03 °/h, and the relative improvement rate stays mostly below 2%, signifying entry into a deep diminishing marginal return zone.

Quantitative statistics reveal that the absolute improvement from 60 to 63 IMUs is merely 0.02 °/h with a relative gain of 2.00%, while the figures for 63 to 66 IMUs are 0.03 °/h and 3.06%, respectively. After 60 IMUs, no adjacent interval delivers an absolute improvement exceeding 0.03 °/h, and the relative improvement rate consistently falls below 4% with continuous attenuation. Accordingly, 60 IMUs can be regarded as the onset of the performance saturation region.

Geometric knee point detection is further performed on the normalized sequence by calculating the perpendicular distance from each data point to the line connecting the first and last points. The maximum distance of 0.3970 corresponds to N = 21, indicating that the central transition point where the curve shifts from steep descent to gentle decay lies around 21 IMUs. Combined with the continuous variation trend of marginal improvement, the performance knee point interval is identified as 21–30 IMUs. Within this range, the absolute improvement from adding every three IMUs gradually falls from 0.10 °/h to 0.07 °/h, and the relative improvement rate drops from approximately 6% to around 4.7%, with marginal benefits already weakening substantially.

From the perspective of engineering costs, doubling the IMU quantity from 30 to 60 only improves the bias stability from 1.42 °/h to 1.00 °/h, a relative enhancement of roughly 29.6%. Meanwhile, power consumption, volume, overhead of synchronous channels, and real-time computational load of the fusion algorithm nearly double linearly. Non-ideal factors such as calibration complexity and inconsistency of temperature drift also rise sharply.

Balancing accuracy improvement and resource requirements, 21–30 IMUs can be regarded as a reasonable engineering configuration range, with approximately 30 IMUs providing a favorable trade-off between performance and cost. If a moderate relaxation of the accuracy requirement is acceptable, approximately 21 IMUs may provide higher cost-effectiveness. For applications with stricter accuracy requirements and sufficient hardware resources, the array size may be moderately extended to 30–40 IMUs. Further increasing the array size beyond approximately 60 IMUs provides only limited marginal improvement under the conditions considered in this study.

## 7. Conclusions

This paper investigates the data fusion problem of multi-MEMS gyroscope arrays and comparatively studies four methods: numerical averaging, weighted least squares, direct estimation Kalman filtering, and indirect estimation Kalman filtering. The performance of these methods is evaluated through theoretical analysis, simulations, static experiments, and dynamic turntable experiments, while the state estimation characteristics and observability of the two Kalman filtering methods are also analyzed.

The simulation and static experimental results show that when the IMUs have similar error characteristics, the performance differences among the four methods are relatively small. When the error characteristics differ significantly, constructing fixed weights based only on a single statistical indicator may lead to weight mismatch, and the performance of weighted least squares may even be inferior to that of numerical averaging. In the dynamic experiment, the data fused by the four methods are used for PSINS inertial navigation, with the final horizontal position drift adopted as the primary evaluation metric. The results show that the direct estimation Kalman filter achieves the best performance, followed by the indirect estimation Kalman filter, numerical averaging, and weighted least squares. Overall, the two Kalman filtering methods exhibit better dynamic navigation performance, among which the direct estimation Kalman filter provides better overall performance under the experimental conditions of this study.

The array-size analysis further shows that increasing the number of IMUs can improve the bias stability of the array, but the performance gain gradually decreases. Considering accuracy, computational burden, hardware cost, power consumption, and system complexity, approximately 21–30 IMUs can be regarded as a reasonable range for engineering configuration.

Future work will further investigate adaptive weighting, robust Kalman filtering, and multi-source information-assisted estimation. On the basis of the current multi-gyroscope array fusion framework, additional sensor information from accelerometers, high-accuracy IMUs, or GNSS may be introduced to provide new observational constraints for gyroscope error estimation. Joint modeling and fusion of different sensor types will also be investigated to further improve the fusion accuracy and robustness of MEMS inertial measurement arrays under complex dynamic conditions.

## Figures and Tables

**Table 1:** Simulation parameters for IMU models.

Simulation 1		Simulation 2	
IMU ID	Std (deh)	IMU ID	Std (deg/h)
IMU A1	0.3	IMU A2	0.1
IMU B1	0.3	IMU B2	0.2
IMU C1	0.3	IMU C2	0.3
IMU D1	0.3	IMU D2	0.4

**Table 2:** Simulation results.

Simulation 1		Simulation 2	
Fusion Method	Std (deg/h)	Fusion Method	Std (deg/h)
Numerical Averaging	0.15	Numerical Averaging	0.14
Weighted Least Squares	0.15	Weighted Least Squares	0.08
Direct Estimation Kalman Filter	0.01	Direct Estimation Kalman Filter	0.01
Indirect Estimation Kalman Filter	0.15	Indirect Estimation Kalman Filter	0.14

**Table 3:** Bias stability parameters for IMU 1–IMU 8.

Group 1 IMUs	Bias Stability (deg/h)	Group 2 IMUs	Bias Stability (deg/h)
IMU 1	3.26	IMU 5	1.35
IMU 2	3.13	IMU 6	2.22
IMU 3	3.20	IMU 7	3.09
IMU 4	3.09	IMU 8	4.35

**Table 4:** Experimental results of four fusion methods.

Experiment 1		Experiment 2	
Fusion Method	Bias Stability (deg/h)	Fusion Method	Bias Stability (deg/h)
Numerical Averaging	1.54	Numerical Averaging	1.39
Weighted Least Squares	1.54	Weighted Least Squares	1.10
Direct Estimation Kalman Filter	1.54	Direct Estimation Kalman Filter	1.10
Indirect Estimation Kalman Filter	1.54	Indirect Estimation Kalman Filter	1.10

**Table 5:** Standard deviation and RMSE of four fusion methods in Experiment 1 and Experiment 2.

Simulation Case 1			Simulation Case 2		
IMU ID	Std (deg/h)	Repeatability (deg/h)	IMU ID	Std (deg/h)	Repeatability (deg/h)
IMU E1	0.1	0.1	IMU E2	0.1	0.4
IMU F1	0.2	0.1	IMU F2	0.2	0.3
IMU G1	0.3	0.1	IMU G2	0.3	0.2
IMU H1	0.4	0.1	IMU H2	0.4	0.1

**Table 6:** Simulation results of two groups.

Simulation Case 1 Results			Simulation Case 2 Results		
Fusion Method	Std (deg/h)	RMSE (deg/h)	Fusion Method	Std (deg/h)	RMSE (deg/h)
Numerical Averaging	0.14	0.15	Numerical Averaging	0.14	0.19
Weighted Least Squares	0.08	0.11	Weighted Least Squares	0.08	0.29
Direct Estimation Kalman Filter	0.001	0.001	Direct Estimation Kalman Filter	0.001	0.001
Indirect Estimation Kalman Filter	0.14	0.15	Indirect Estimation Kalman Filter	0.14	0.19

**Table 7:** Bias stability of IMU1–IMU8.

Group 1 IMUs	Bias Stability (deg/h)	Group 2 IMUs	Bias Stability (deg/h)
IMU 1	3.26	IMU 5	1.35
IMU 2	3.13	IMU 6	2.22
IMU 3	3.20	IMU 7	3.09
IMU 4	3.09	IMU 8	4.35

**Table 8:** Bias stability and RMSE of four fusion methods in Experiment 1 and Experiment 2.

Experiment 1			Experiment 2		
Fusion Method	Bias Stability (deg/h)	RMSE (deg/h)	Fusion Method	Bias Stability (deg/h)	RMSE (deg/h)
Numerical Averaging	1.54	0.21	Numerical Averaging	1.39	0.17
Weighted Least Squares	1.54	0.21	Weighted Least Squares	1.10	0.23
Direct Estimation Kalman Filter	1.54	0.0023	Direct Estimation Kalman Filter	1.10	0.019
Indirect Estimation Kalman Filter	1.54	0.21	Indirect Estimation Kalman Filter	1.10	0.17

## Data Availability

The original contributions presented in this study are included in the article. Further inquiries can be directed to the corresponding author.
